# A Social-Ecological Model Exploring Gestational Diabetes Mellitus Screening Practices Among Antenatal Health Care Providers

**DOI:** 10.1177/10901981241232651

**Published:** 2024-02-26

**Authors:** Emma Ruby, Sarah D. McDonald, Howard Berger, Nir Melamed, Jenifer Li, Elizabeth K. Darling, Michael Geary, Jon Barrett, Beth Murray-Davis

**Affiliations:** 1 McMaster University, Hamilton, Ontario, Canada; 2 University of Toronto, Toronto, Ontario, Canada; 3 Rotunda Hospital, Dublin, Ireland

**Keywords:** counseling, general terms, health behavior, health communications, maternal child health, patient-provider communication, pregnancy, qualitative methods, screening

## Abstract

Gestational diabetes mellitus (GDM) is associated with adverse health outcomes for the pregnant individual and their baby. Screening approaches for GDM have undergone several iterations, introducing variability in practice among healthcare providers. As such, our study aimed to explore the views of antenatal providers regarding their practices of, and counseling experiences on the topic of, GDM screening in Ontario. We conducted a qualitative, grounded theory study. The study population included antenatal providers (midwives, family physicians, and obstetricians) practicing in Hamilton, Ottawa, or Sudbury, Ontario. Semi-structured telephone interviews were conducted and transcribed verbatim. Transcripts were analyzed using inductive coding upon which codes, categories, and themes were developed to generate a theory grounded in the data. Twenty-two participants were interviewed. Using the social-ecological theory, we created a model outlining four contextual levels that shaped the experiences of GDM counseling and screening: Intrapersonal factors included beliefs, knowledge, and skills; interpersonal factors characterized the patient-provider interactions; organizational strengths and challenges shaped collaboration and health services infrastructure; and finally, guidelines and policies were identified as systemic barriers to health care access and delivery. A focus on patient-centered care was a guiding principle for all care providers and permeated all four levels of the model. Patient-centered care and close attention to barriers and facilitators across intrapersonal, interpersonal, organizational, and policy domains can minimize the impact of variations in GDM screening guidelines. Among care providers, there is a desire for additional skill development related to GDM counseling, and for national consensus on optimal screening guidelines.

Gestational diabetes mellitus (GDM) is one of the most common complications of pregnancy, and rates have been on the rise in Canada, from 4.0% in 2004 to 7.0% in 2014 (Metcalfe et al., 2017). Variations in screening approaches, diagnostic thresholds, and increased incidence of delayed childbearing, obesity, and excess gestational weight gain have contributed to a rising incidence over the last 20 years (Ben-Haroush et al., 2004; Berger et al., 2019; Feig et al., 2014; Johns et al., 2018).

Currently in Canada, two approaches to screening are endorsed by Diabetes Canada (DC) and the Society of Obstetricians and Gynecologists of Canada (SOGC): the “preferred” two-step method of a non-fasting, 50-gram oral glucose challenge test (50 g OGCT) followed by a fasting 75-gram oral glucose tolerance test (75 g OGTT) upon abnormal results, as well as an “alternate” method of the one-step, fasting 75-g OGTT (Berger et al., 2019; Diabetes Canada, 2022; Mussa et al., 2021). A randomized controlled trial, comparing the incidence of GDM between screening approaches, revealed a nearly doubled incidence rate of GDM in the group who underwent a one-step 75-g OGTT when compared to the group who was administered a 50-g OGCT followed by a 100-g OGTT (16.5% vs. 8.5%, respectively) (Hillier et al., 2021).

Globally, there is a lack of consensus regarding an optimal GDM screening approach (Berger et al., 2019; Diabetes Canada, 2022; International Association of Diabetes and Pregnancy Study Groups Consensus Panel, 2010; Lu et al., 2016). For example, the approaches endorsed by DC and the SOGC differ from that of the International Association of the Diabetes and Pregnancy Study Groups, which supports the use of the one-step 75-g OGTT with lower diagnostic cutoff values (Berger et al., 2019; Diabetes Canada, 2022; International Association of Diabetes and Pregnancy Study Groups Consensus Panel, 2010). There has also been debate over whether to practice universal- or risk-based GDM screening. The SOGC and DC have shifted from a selective screening approach employed through the 1990s and early 2000s, to now recommending universal screening (Mussa et al., 2021).

From a health care provider perspective, there is some evidence suggesting feelings of uncertainty and frustration with the ever-changing guidelines (Bandyopadhyay, 2021; Green et al., 2020; Tait Neufeld, 2014; Timm et al., 2021). While trying to navigate the various treatment pathways for GDM, care providers reported concerns about restrictions to their scope of practice and poor communication and collaboration across clinical settings which were seen to contribute to lack of understanding and unnecessary repetition care (Timm et al., 2021). Other challenges faced by care providers included lack of time, training, and resources to provide culturally safe and personalized GDM counseling (Bandyopadhyay, 2021; Tait Neufeld, 2014). This was seen to limit the ability to individualize treatment plans to specific patient populations (Bandyopadhyay, 2021).

We sought to understand how the lack of consensus around GDM screening guidelines influenced care provider counseling and patient decision-making, with specific attention to similarities and differences in approaches across health care professions and urban and rural locations in Ontario, Canada. Our findings from our first phase of the study identified that patients, regardless of antenatal care provider or geographic region, received similar counseling and recommendations suggesting that, despite the lack of consensus on optimal screening approaches, there may be less variation at the patient level (Ruby et al., 2023). The aim of the phase of the study reported here was to investigate these findings further by exploring the experiences of GDM counseling and screening from the perspectives of antenatal providers practicing in the same geographic regions in Ontario.

## Methods

We conducted a qualitative, grounded theory study with antenatal care providers. Ethical approval was obtained from the Hamilton Integrated Research Ethics Board (HiREB Project: 7916).

Midwives (MWs), family physicians (FPs), and obstetricians (OBs) practicing within Hamilton, Ottawa, or Sudbury, Ontario, Canada, were eligible to participate. Convenience and purposive sampling were used for recruitment, using social media and posters within the community. The geographic regions identified for inclusion were selected to increase variability of the sub-populations across Ontario and to utilize existing contacts to assist in the recruitment process. A minimum of three participants from each professional group and from each geographic region was identified as the desirable sample size (totaling 27 participants) but with the intention of continuing recruitment until we reached saturation.

Semi-structured interviews occurred between March 2020 and December 2020 and were conducted over the telephone for approximately 30–45 minutes. The interview guide was developed by our research team and utilized during the interview process, using a mix of open- and closed-ended questions. There were no deviations from the interview guide between March and December of 2020. This ensured each participant’s responses were valued equally in the case of any adaptations to health screening services during the COVID-19 pandemic.

In keeping with grounded theory, data analysis began at the same time as data collection, to make use of the iterative process of constant comparison and adaptation to the participant’s responses (Chun Tie et al., 2019; Corbin & Strauss, 1990). Interviews were transcribed verbatim and entered into NVivo 11 software (QSR International Pty Ltd., 2015). Data analysis began with open coding. Initial open coding of three transcripts was completed by three independent researchers to ensure consistency and inter-rater reliability in the coding process (O’Connor & Joffe, 2020). Upon completion of open coding, codes were then grouped to form axial codes. Axial coding provided a framework from which the open codes could be synthesized into hierarchically structured categories (Fereday & Muir-Cochrane, 2006; Sbaraini et al., 2011; Scott & Medaugh, 2017; Walker & Myrick, 2006). Finally, during the selective coding process, further grouping of axial codes formed themes that, when brought together, generated a theory grounded in the data (Walker & Myrick, 2006).

Interim analyses were shared by the researchers at debrief meetings, and the principal investigator reviewed the coding at each stage of analysis to ensure scientific rigor and sound methodology (O’Connor & Joffe, 2020). The research team comprised students and experts from a variety of disciplines, including midwifery, maternal fetal medicine, and health research methodology. Investigator triangulation was used to review, validate, and come to agreement on disputed codes between researchers (Carter et al., 2014). These approaches were employed to minimize bias, strengthen credibility, and add breadth to the emerging phenomena (Carter et al., 2014).

## Results

A total of 22 participants were included. Due to the small number of participants in the included regions and the risk of identifying individuals, demographic characteristics were not obtained for participants aside from the geographic location in which they practiced (Table 1).

**Table 1.** Participant Distribution According to Geographic Location and Professional Identity.

Geographic location	Family physicians (*n* = 6)	Midwives (*n* = 10)	Obstetricians (*n* = 6)
Hamilton, ON	3	3	3
Ottawa, ON	2	4	2
Sudbury, ON	1	3	1

**Figure 1.**
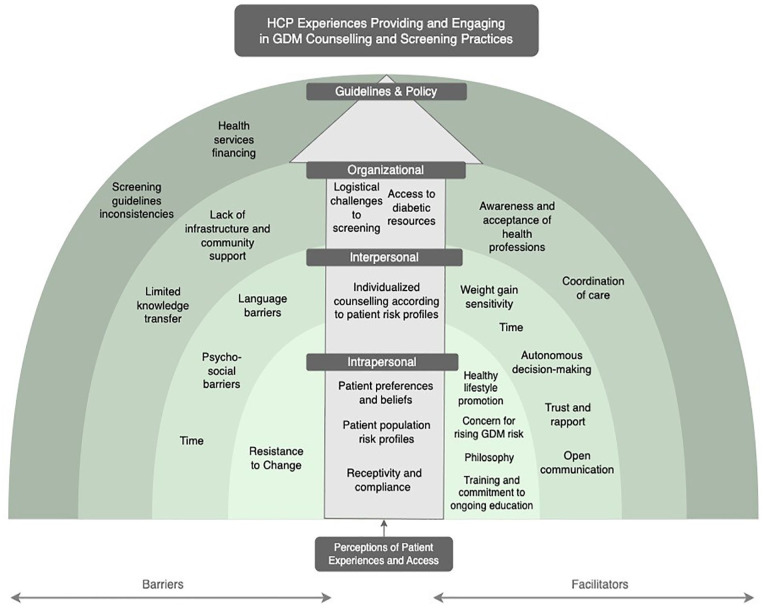
Socio-Ecological Model Highlighting the GDM Counseling and Screening Experiences of Antenatal Health Care Providers. *Note.* HCP = Health Care Provider; GDM = gestational diabetes mellitus.

Using the social-ecological framework developed by Bronfenbrenner, we developed a model outlining four contextual levels—intrapersonal, interpersonal, organization and guidelines and policy—that influenced the experiences for antenatal care providers regarding GDM counseling and screening (Figure 1) (Bronfenbrenner, 1977). Originally a framework to understand human development, Bronfenbrenner’s social-ecological model has been widely used and adapted by various researchers to highlight different ecosystems related to an individual’s life experiences (Kilanowski, 2017). The framework typically depicts the innermost systems being directly experienced at the individual level, with expansion outwards as the systems become more abstract and wide-reaching (Kilanowski, 2017). Our adapted model is categorized according to barriers (left) and facilitators (right) within each contextual level. In addition, an ascending arrow that transcends all contextual levels was included to reflect patient centeredness as a key pillar in providers’ experiences.

### Intrapersonal

The intrapersonal level relates to the knowledge, skills, values, and attitudes of participants, including their beliefs, as well as those of their patients. Prior training and commitment to ongoing education was highlighted by several participants as facilitating knowledge and improved GDM management. Examples included engaging in medical education programs, sub-specialized training, exposure through direct patient engagement, personal interest pursuits, and commitment to keeping updated on research evidence.

The philosophy of care for each provider informed the approaches to care, how they engaged with their patients, and how they navigated their role in health systems. Examples of guiding principles included the importance of universal screening, patient autonomy, research-to-practice knowledge translation, ongoing commitment to quality assurance, informed consent, and informed choice:

I think that these differences often have less to do with the particular issue, in this case, gestational diabetes mellitus, than how we approach issues in general. So, because mine is an informed choice approach, I know that not everybody practices that way. (FP2).

Concerns about rising rates of GDM were expressed by several participants. These sentiments reflected a heightened awareness of the implications and risks associated with a GDM diagnosis for the health of pregnant individuals and their babies, as well as the implications for the functioning of health systems. Similarly, healthy lifestyle promotion was a facilitator for providing GDM counseling. For example, many participants were averse to using body mass index (BMI) as an indicator of health, with the belief that variations in bodily responses to pregnancy should be normalized, not stigmatized: “I have a high BMI myself, and I don’t like the idea that we approach women presuming that they have health problems as a result of their BMI.” (MW3).

Some participants articulated resistance to change regarding GDM guidelines. This was characterized by the belief that there would be further revisions of GDM screening guidelines in the future, or even shifting of the guidelines back to a previous version. Providers described doubts about the evolving nature of evidence and a reluctance about changing their practice given inconsistent evidence for optimal GDM practice standards:

I think when guidelines do change a lot, there might be a thought that “it’s going to eventually come around, kind of like trends. It’s going to be in style again the way I’m doing it, so I may as well just keep doing it the way I’m doing it.” (MW1)

### Interpersonal

Interpersonal relations between professionals and their patients included factors that influenced the quality and quantity of interactions during the provision of GDM counseling and screening.

Time to provide counseling during appointments was presented as both a barrier and facilitator across all professions. Notable differences existed between professions, particularly for MWs when compared to OBs and FPs. The majority of MWs acknowledged that their model of practice and smaller caseload provided them with more time for client engagement. However, many physicians expressed that limited time impeded the quality of counseling they could provide (Table 2).

**Table 2.** “Time” Expressed as a Facilitator and Barrier With Exemplar Quotes.

Facilitator	“As a midwife, I have half-hour appointments. I have one-hour appointments. I have multiple opportunities to talk to people about GDM screening and just overall health teaching.”—(MW2).
Barrier	“My counselling is limited very much by time. So, appointments are not long, and we see a lot of people in a day. . .. It’s hard to have long, involved conversations with everyone and see a patient population”—(OB1). “Time is always a barrier as family doctors are not funded fairly, haven’t received an upward increment in our billings in decades. There is no billing code that exists to represent the time and energy to exhaustively counsel and lecture a growing population of people who are at risk or have GDM.”—(FP6)

*Note.* GDM = gestational diabetes mellitus; MW = midwives; OB = obstetricians; FP = family physicians.

The ability for patients to understand the information provided was also described as a barrier. Limited patient understanding was attributed to several factors, such as patient education levels, psycho-social circumstances impacting motivation for, and accessibility of, GDM services, language barriers, and a lack of access to translator services:

Interpretation services are a big [barrier]. It is not ideal to have a person on a speaker phone when you don’t necessarily know how well the client is understanding, and of course, with the time it takes to translate. (MW1).

Engagement in discussions regarding weight gain, diet, and exercise in a sensitive manner was a notable positive factor that impacted the provision of meaningful GDM information. Many participants expressed awareness of the importance of sensitive language when discussing topics related to food, diet, and weight management:

I think it is a difficult conversation, right? We hear from clients who have a higher BMI, right? The BMI is often the elephant of the room. So being aware of that and trying to do your best about language and sensitivity around it, but while not avoiding those difficult conversations. (MW6).

Most participants highlighted the importance of respecting patient autonomy. MWs related this model of decision-making to their profession’s philosophy of informed choice, “I feel like people have the right to make their own decisions, and I feel like the guidelines say that there is universal screening . . . You know, people have a right to screen or not.” (MW8). Likewise, many obstetric and physician participants expressed a similar approach to decision-making: “Sometimes I’ll repeat the screening if that’s the risk, but if somebody says, ‘I don’t want screening,’ that’s totally fine. It’s their choice, and I respect their decision.” (OB4). Nearly every participant indicated that they believed it was important to offer GDM screening to all their patients, regardless of their risk factors, compliance, or motivation for screening uptake.

Trust and rapport were catalysts for screening compliance and were typically coupled with other facilitators, such as unrestricted time and alignment of philosophical values between professionals and patients. In addition, open communication between providers and their patients created a sense of ease, comfortability, and accessibility. Communication techniques that the care providers reported using included ensuring language were comprehensible and accessible to patients and providing information and resources, through counseling, handouts and pamphlets, websites, or referrals, to ensure patients could make an informed decision about their health care.

### Organizational

Interactions with other professionals, communities, health sectors, and regulatory bodies were identified as organizational factors that influenced care provider experiences. A lack of sufficient community services and infrastructure was most noted by those practicing in Northern areas. Contributing factors included limited lab locations, reduced lab capacities, underserved GDM programs, limited staffing, and a wide catchment area. One participant summarized these factors when they stated:

Many of our clients wouldn’t do it if they had to go to a lab, because there weren’t many labs. They had to drive to one of the cities or small towns and use the hospital lab, usually. So, you know, the parking, the sickness and germs in hospitals, everything made it kind of much more likely that they would say no [to screening]. (MW7).

Likewise, knowledge transfer, defined as the sharing of practice guidelines and operating protocols across clinical settings, was lacking. Participants described how sometimes there was inconsistency between different clinical settings regarding GDM screening, management, and follow-up. This created confusion and frustration. For example, one MW described how the interpretation of GDM screening cutoffs was different at the two hospitals where she had privileges. This would mean that at one hospital site, a client would be diagnosed with GDM, while at the other hospital, they would be seen as a screen negative. These variations were difficult to explain to patients and created interprofessional tensions:

Navigating [GDM guidelines] as someone who operates adjacent to that system, right? How can I justify, for example, choosing to not send a client to one hospital because they would have gestational diabetes per that hospital’s protocol? How can I feel confident in making those decisions if I’m faced with the scrutiny of other healthcare providers? (MW3).

Service coordination with other community services was seen by participants as an important facilitator that improved the provision of GDM care. For example, having several specialists accessible to patients upon diagnosis of GDM, such as dieticians, endocrinologists, and OBs, was particularly beneficial. In addition, several participants expressed gratitude for working within a shared-care model, collaborating with others to share expertise and provide comprehensive care.

I surround myself with lots of colleagues who do OB. I don’t work solo, so I feel reasonably confident in my abilities to at least screen for it and counsel for it and manage in a shared arrangement once they become diabetic. (FP5)

### Guidelines and Policy

Participants highlighted how guidelines and policies influenced professional roles (summarized in Table 3). Financial restrictions within the health care system were articulated as a barrier. Some participants recalled their patients declining GDM screening due to potential financial burden if diagnosed with GDM without insurance coverage. For example, one physician described a common scenario in which their patients would be provided a complimentary “starting” package for GDM management but could not afford to maintain this due to a lack of funding for testing equipment:

**Table 3.** Inconsistent Components of Screening Recommendations & Exemplar Quotes.

Approach to testing	What is clear is that if they fail the 50 [gram OGCT], then we go to 75 [gram OGTT], and I think that the actual criteria for whether they failed the 50 or 75 g, that’s clear from the guidelines . . . it’s just some people are confused about which test they should be using. (FP3).
Diagnostic thresholds	There’s an old set of thresholds and then there’s a new set of thresholds and they’ve even waffled on that by saying, ‘individual centers can do what they want.—(OB2)
Care plan & management	The lack of consistency in management after diagnosis, I have to say is a bit of a frustration . . . Our community standard and the variance across the providers in the communities: midwives, obstetricians, family doctors, is huge—(MW9).

*Note.* OGCT = oral glucose challenge test; OGTT = oral glucose tolerance test; FP = family physicians; OB = obstetricians; MW = midwives.

The other thing, unfortunately in Canada right now is cost. I mean we’re able to give people glucometers and a little sample of test strips and lancets which is 10 of them in a package . . . We try and minimize the testing so that it’s affordable. But that’s a huge barrier and it doesn’t matter even if [the patient] has OHIP benefits, it does not cover test strips and lancets. That’s just appalling in our country. (OB2)

Screening guideline inconsistencies presented considerable challenges for participants. Frustrations arose due to inconsistencies regarding the optimal test to recommend, differences in diagnostic test thresholds, and variations between professionals and between communities.

### Patient Experiences and Access to Screening

Care providers’ perceptions of the patient experience was situated in the middle of our model and transcended all contextual levels. The placement of the arrow was intended to signify the interconnected nature of the patient care experience in the experiences of care providers.

Participants described ways in which patient preferences and beliefs were sometimes a barrier to screening, particularly if patients preferred to avoid non-essential medical interventions or perceived themselves as being minimal risk, therefore impeding motivation for screening. The care provider-patient relationship influenced the approach to counseling. Care providers described times when they adapted GDM counseling to be more person-centered. The most common example of this was when they would suggest early trimester screening for higher risk patients:

If they have any risk factors—usually at the first prenatal visit, if there’s any risk factors such as obesity, or family history, or ethnicity, then I would counsel them—I would suggest that we screen earlier in the pregnancy. (FP3).

Care providers also discussed how they tailored their approach based on community-specific risk factors such as the risk profiles of patient populations according to the geographic region and whether the care provider felt able to manage care for higher risk patients. Most participants indicated that the majority of their patients accepted screening; however, there were differences in screening compliance according to the adequacy of infrastructure and services available in the communities in which they practiced: “I don’t really find anyone outright declining the test to be honest, but there are people who seem to have a difficult time getting it done and those are often folks from underserviced populations.” (FP1).

Care providers perceived barriers for patients at the organizational level including logistical challenges to accessing screening. Common barriers articulated by participants included transportation challenges, time commitment, child care scheduling, hesitation due to COVID-19 exposure risk, delays in screening access due to COVID-19, inconvenient lab location, fasting coordination challenges, cost, and limited lab capacities. These challenges to accessing screening were noted to vary considerably across geographic regions, with rural or remote areas experiencing greater barriers than urban areas.

## Discussion

This study explored the practices of, and counseling on the topic of, GDM screening by antenatal care providers, specifically MWs, OBs, and FPs in Hamilton, Sudbury, and Ottawa, Ontario. The goal of this paper was to provide a qualitative analysis to explore care providers views and attitudes toward GDM screening in a Canadian context.

The socio-ecological framework was used to organize our findings and to develop our theoretical model (Bronfenbrenner, 1977). Depicting our findings using this approach of intrapersonal, interpersonal, organizational, and guidelines/policies provided a means to highlight the multiple interacting determinants that impact behaviors for individuals, both providers and patients, engaging in health care services (Glanz et al., 2015; Golden & Earp, 2012). At the center of our model, and transcending all levels, was the patient experience. This strong focus on patient-centered approaches to GDM counseling and screening practices was evident across all professions and regions. We found that care providers rarely expressed perspectives that were isolated from their patient’s experiences, goals, or interests. Thus, it was integral to our study that the patient experience act as a guiding pillar.

Participants described how intrapersonal factors such as personal values and professional philosophies shaped their perspective. Personal beliefs and preferences further shaped their approaches to care and highlighted topics of consideration based on the patient populations that they served, including weight gain, diet, and healthy lifestyle promotion. At the interpersonal level, we identified that supporting autonomy and facilitating patient comprehension, while maintaining effective communication, were key determinants of the decision-making process, and they often impacted the level of comfort and confidence the patient felt in their health care decisions. The impact of intrapersonal factors on counseling has been reflected in previous research, which emphasized similar challenges to those expressed in our study, including issues with personalizing care plans, communicating health care goals between providers and patients, and maintaining consistent follow-up with patients to encourage screening (McCloskey et al., 2019; Nielsen et al., 2014; Tierney et al., 2015).

Organizational factors that arose from the data were indicative of both strengths and challenges in care coordination. This included the ability to collaborate with care providers across health sectors and accessibility of community health services (Meloncelli et al., 2019; Persson et al., 2011; Timm et al., 2021). For example, OBs report engaging in discussions regarding short-term implications of GDM, but they rely on other health care providers for long-term risk counseling (Timm et al., 2021). Systemic, policy-level factors such as the inconsistencies of guidelines and gaps in fundings and access to care were seen as barriers, impeding participants’ ability to navigate GDM counseling and screening with ease and confidence.

One of the aims of this study was to compare the differences, if any, in care practices between antenatal care providers. The most prominent distinctions between professions related to patient autonomy, time spent directly with patients, and professional role integration and coordination owing to both professional philosophies and scope of practice. We also sought to understand any differences between urban versus rural regions. Challenges in accessing screening and limited lab capacities were noted among those practicing in more northern communities. This is consistent with current literature that highlights challenges with understaffing, limited budgets, lack of time, and a lack of resources for GDM care in rural areas (Jacklin et al., 2017; Oza-Frank et al., 2014; Sayakhot & Carolan-Olah, 2016; Sharma et al., 2016; Tait Neufeld, 2014).

Our study was one of the first to explore and compare the experiences between antenatal care providers in a Canadian context. This study highlighted the direct impact that guidelines have on patients and providers, through the identification of barriers and facilitators at multiple contextual levels. The findings from our study contribute meaningful, provider-centered insight into the strengths and challenges related to GDM screening and counseling, findings for which the existing body of evidence has historically been lacking. Our study provides an important population perspective which should be conveyed to, and considered by, national organizations that promote clinical practice guidelines. Furthermore, by organizing our findings according to contextual levels, future research directions and changes to clinical practice guidelines may be implemented using a tiered approach similar to what we have presented in this study.

The strengths of our study included the multidisciplinary nature of the research team, the range of geographic regions used for recruitment, and the inclusion of three different health professions. A limitation of our study was potential selection bias, given that those who volunteered to participate were likely willing to do so based on their personal beliefs about, and experiences with, the research topic (Newington & Metcalfe, 2014). Likewise, our sample may not represent the general population, as these individuals likely had stronger opinions about the topic than those who chose not to participate (Newington & Metcalfe, 2014). The COVID-19 pandemic may have also presented possible selection bias due to early cessation of recruitment for this study as a result of uncertainty in restrictions and research protocols. In addition, we acknowledge that the COVID-19 pandemic likely presented a unique set of challenges and changes related to health screening provision that may have influenced our findings. Finally, language was a limitation in our study, given that the interviews were only conducted in English (Willis et al., 2021).

## Conclusion

Our findings indicated that GDM counseling and screening practices are largely influenced by direct engagement with patients and a desire to provide patient-centered care. These interactions are further impacted by individual, interpersonal, organizational, and systemic factors that create discrepancies in expectations, protocols, and responsibilities that care providers must navigate in their professional role. Close attention to barriers and facilitators across these domains may minimize the impact of variations in GDM screening guidelines. Among care providers, there was general interest in further skill development to aid GDM counseling and a desire for consensus on optimal screening guidelines.
